# Acute Stress Modulates Feedback Processing in Men and Women: Differential Effects on the Feedback-Related Negativity and Theta and Beta Power

**DOI:** 10.1371/journal.pone.0095690

**Published:** 2014-04-22

**Authors:** Stella Banis, Linda Geerligs, Monicque M. Lorist

**Affiliations:** 1 Department of Experimental Psychology, University of Groningen, Groningen, The Netherlands; 2 BCN Neuroimaging Center, University of Groningen, Groningen, The Netherlands; Erasmus University Rotterdam, Netherlands

## Abstract

Sex-specific prevalence rates in mental and physical disorders may be partly explained by sex differences in physiological stress responses. Neural networks that might be involved are those underlying feedback processing. Aim of the present EEG study was to investigate whether acute stress alters feedback processing, and whether stress effects differ between men and women. Male and female participants performed a gambling task, in a control and a stress condition. Stress was induced by exposing participants to a noise stressor. Brain activity was analyzed using both event-related potential and time-frequency analyses, measuring the feedback-related negativity (FRN) and feedback-related changes in theta and beta oscillatory power, respectively. While the FRN and feedback-related theta power were similarly affected by stress induction in both sexes, feedback-related beta power depended on the combination of stress induction condition and sex. FRN amplitude and theta power increases were smaller in the stress relative to the control condition in both sexes, demonstrating that acute noise stress impairs performance monitoring irrespective of sex. However, in the stress but not in the control condition, early lower beta-band power increases were larger for men than women, indicating that stress effects on feedback processing are partly sex-dependent. Our findings suggest that sex-specific effects on feedback processing may comprise a factor underlying sex-specific stress responses.

## Introduction

Several mental and physical disorders show sex-specific prevalence rates. For example, men have higher rates of addiction disorders and cardiovascular diseases, whereas women are more susceptible to depression and anxiety disorders and autoimmune diseases (see for reviews, [1], [2]). Physiological responses to stress have been proposed to play an important role in the pathogenesis of these disorders. This raises the possibility that sex-specific prevalence rates are at least partly due to sex-specific stress responses [1]. Nevertheless, the neural mechanisms underlying these effects are largely unknown. Increasing evidence suggests that particular stress-related disorders, such as mood disorders and drug addiction, are associated with abnormal feedback processing [3], [4]. In the present study, we therefore focused on feedback-related neural activity in men and women.

Recent research has revealed that exposure to acute stress alters decision-making behavior by modulating risk-taking behavior [5]–[9], and by affecting learning from feedback. A number of studies, for example, have found that stress impairs learning from positive feedback [10] or negative feedback [11]. However, a recent study found that the effects of stress on reward learning (learning from seeking reward) or punishment learning (learning from avoiding punishment) depend on the punishment sensitivity and stress reactivity of the participant [12]. This indicates that stress effects on feedback learning are not necessarily negative and depend on individual characteristics.

Feedback processing and feedback learning are of crucial importance to adaptive decision making. Although there is some knowledge about the behavioral effects of stress on feedback learning, knowledge about the neural underpinnings of these stress effects is scarce. Brain regions that are associated with feedback processing and learning (e.g., the ventral striatum, medial frontal cortex (MFC), orbitofrontal cortex (OFC), and lateral prefrontal cortex (PFC)) have been shown to be sensitive to stress-induced changes (see for reviews, [13], [14]), supporting the notion that stress influences feedback processing and learning. In addition, recent fMRI studies have reported reduced responses of these brain areas to monetary outcomes under stress [15], [16]. In the current study, our first aim was to gain more insight into the impact of acute stress on feedback processing in men and women on a neural level, applying electroencephalography (EEG).

Studies using EEG have identified an ERP component that is elicited in response to external feedback: the feedback-related negativity (FRN). The FRN is a negative ERP component, which peaks between 250 and 300 ms after feedback delivery, is maximal over frontocentral scalp sites, and is larger in amplitude following negative compared to positive feedback [17], [18]. The major contributors to the FRN are probably located in the MFC [19]. The specific function of the MFC in feedback processing has been debated: evaluating decision outcomes to guide reward-seeking behavior [20], [21]; or monitoring for response conflict (the simultaneous activation of competing responses), with conflict detection leading to compensatory adjustments in control [22]. Botvinick [23] has tried to reconcile these two perspectives, proposing that these different functions may be part of a general learning system biasing behavioral decision making toward cognitively efficient strategies. Emerging evidence points at functional interactions between the MFC and other prefrontal cortical regions [19], [24], [25]. Monitoring-related activity in the MFC appears to serve as a signal that engages regulatory processes in the lateral PFC to implement behavioral adjustments.

In a previous study [26], we investigated the impact of acute noise stress on the FRN in men, and whether effects depended on stressor predictability. Participants performed a gambling task in a control and a stress condition with either a predictable or unpredictable noise stressor. FRN amplitude was measured in different ways, either neglecting or correcting for overlap with other components. We found that acute noise stress specifically modulated valence and magnitude effects on the FRN, with smaller effects in the stress relative to the control condition, although evidence differed between measures as to whether valence and/or magnitude were processed differently. We interpreted these findings as a stress-induced impairment of feedback processing. Stressor predictability added little to the explanation of effects. In the current study, we further examined the impact of acute noise stress on feedback processing, using the same gambling task in combination with the unpredictable noise stressor, but now in both sexes.

Until recently, most EEG studies on feedback processing have focused on the FRN, which only reflects oscillations that are phase-locked to the feedback. Nevertheless, recent research has demonstrated that the analysis of oscillatory activity, which includes both phase-locked and non-phase-locked oscillations, can provide complementary insights into feedback processing [24], [27]. Theta power increases over frontocentral scalp sites have been shown to be larger after negative feedback or losses compared to positive feedback or gains [25], [27]–[32]. Theta-band oscillations in the frontal network have been proposed to play an important role in signaling unfavorable outcomes and implementing adjustments [25]. Findings with regard to beta power are less equivalent. Positive outcomes have been shown to induce increased upper beta-band power over frontocentral sites relative to negative outcomes [25], [27], [32]. However, another study found larger increases for losses relative to gains, in both lower and upper beta-bands [30]. The functional role of beta-band activity in feedback processing is largely unknown. Beta-band oscillations in general have been proposed to signal the tendency to maintain the status quo of the current sensorimotor or cognitive state [33]. In the present study, we used both the FRN and feedback-related changes in theta and beta oscillatory power to investigate feedback processing.

Importantly, a number of studies have found that effects of acute stress on decision-making behavior are sex-dependent. Two studies found increased risk taking in men and decreased risk taking in women, during stress [5], [9]. A later fMRI study by Lighthall et al. [34] could not replicate this sex-dependent stress effect on risk taking, but did find greater reward collection and faster decision speed in males and less reward collection and slower decision speed in females, under stress. In addition, the latter study found that the behavioral sex differences were accompanied by different neural activation patterns; with stress, activation in the dorsal striatum and anterior insula was increased in males but decreased in females [34]. Thus, current knowledge suggests that stress affects decision-making behavior, that these effects are sex-dependent, and that these sex-dependent stress effects on decision-making behavior are associated with sex-dependent brain activity. However, it is not clear whether these differential stress effects on decision making may be linked to differential stress effects on feedback processing. Therefore, the second aim of our study was to examine whether acute stress effects on feedback processing differ between men and women.

In sum, the aim of the present study was twofold. First, we examined whether acute stress alters decision making by affecting feedback processing, as reflected in the FRN and feedback-related changes in theta and beta oscillatory power. Second, we investigated whether stress effects are sex-dependent. Participants performed a gambling task, in a control and a stress condition, while their EEG was recorded. Stress was induced by exposing participants to a noise stressor. Based on the studies described above and our previous study [26], we expected a decreased sensitivity to monetary outcomes under stress, with regard to the FRN. Based on the idea that the FRN and theta-band activity partly reflect similar processes [27], [29], we expected a similar stress effect on theta power. With regard to beta-band activity and to sex differences, we did not formulate hypotheses beforehand.

## Methods

### Participants

Sixty-one healthy, right-handed undergraduate students from the University of Groningen (37 females, mean age  = 21.1 years, range 18–40 years; 24 males, mean age  = 21.9 years, range 18–28 years) participated in the experiment. Data from 16 male participants were also used in a previous study [26]. In this previous study, we examined the impact of acute noise stress on the FRN and whether effects depended on stressor predictability, in men only. During the stress condition, participants were either exposed to a predictable (*n* = 16) or unpredictable noise stressor (*n* = 16). For the current study, we used the unpredictable noise stressor to investigate the impact of acute noise stress in both men and women. We included the 16 male participants from the unpredictable noise stressor group, from our previous study. Subsequently, we measured eight additional male and 37 female participants. Participants reported no evidence of current or past psychiatric disorders, neurological disorders, or head injuries, and were free of CNS-active medication. They were non-smokers, and had normal or corrected-to-normal vision and normal hearing. In addition, female participants had not used hormonal contraceptives within the previous four months. They were not pregnant and had regular menstrual cycling with normal mean cycle length (24–35 days).

To minimize the influence of hormonal fluctuations across the menstrual cycle on feedback processing [35] and stress responsiveness [36]–[38], females participated during the putative midluteal phase of their cycle, between day 10 and day 5 prior to menses [39]. Moreover, it has been shown that during this phase, the hypothalamic-pituitary-adrenal (HPA) stress response to laboratory stressors is relatively comparable to the response in men [36].

Measurement days were scheduled on the basis of self-reported menstrual cycle durations and date of onset of the current cycle. Females with a typical cycle length of 29 days were scheduled on day 20–25 from the first day of menses (day 1). Females with shorter or longer cycles were planned accordingly. Retrospectively, the menstrual cycle phase was verified by tracking backward from the date of onset of the next menses that was reported by the participant. As a result, 13 females were excluded from data analysis. In addition, one female withdrew from participation after five minutes in the stress condition, because she could not endure the noise stressor. Consequently, 23 females completed the experiment, during their midluteal phase (mean age  = 20.4 years, range 18–31 years).

Participants received either course credits or €20 for participation. In addition, they received a monetary bonus depending on their gambling scores, as described below. The study was approved by the Ethical Committee Psychology of the Psychology Department of the University of Groningen, and all participants gave written informed consent.

### Procedure

Participants were instructed to abstain from alcohol and caffeine-containing substances 12 h before the experiment. They arrived at the laboratory at 9.00 a.m. and filled out a questionnaire before application of the electrocap. Participants were seated in front of a computer screen, in a dimly lit, sound-attenuated, electrically shielded cabin. A serial response box was placed under their hands. They completed a gambling task in a stress condition and in a control condition, the order of which was counterbalanced across subjects. Both conditions were separated by a break of 15 minutes, in which subjects remained seated in the cabin.

### Gambling task

Participants performed a simplified version of the gambling task devised by Gehring and Willoughby ([17]; see for technical details, [26]). Each trial started with the presentation of two white cards, one of which the participant selected with a left- or right-hand button-press, according to the location of the chosen card. After the response, the chosen card was highlighted with a thick yellow border, for a randomly varying interval. Then, the card turned into either cyan or magenta, emphasizing the valence of the outcome (gain or loss). Simultaneously, a number (+/− 5 or 25; representing euro cents) appeared on the selected card, indicating how much money was won or lost at the trial. The assignment of the two colors to gains or losses was counterbalanced across participants. This feedback display remained at the screen for 1000 ms, after which the next trial started. At the end of each trial block, participants received additional feedback indicating the amount of money earned during that block. The gambling task consisted of 5 trial blocks of 5-minute duration each, in each experimental condition. Before the experimental trials, there was one practice block of 1-minute duration (excluding instructions).

Each trial outcome was determined randomly by the computer program, with equal weights for all four possible outcomes and with replacement. Participants were not informed about this. Before the practice block, they were instructed about the meaning of the feedback display. They were told that they started the experiment with €5, and that the value of each selected outcome would be added or subtracted, and that they would keep the resulting sum of money. In addition, they were told that they would receive feedback indicating the amount of money earned during the block, at the end of each block. Finally, participants were instructed that their goal was to earn as much money as possible, and that they were free to choose any strategy to achieve this. Our cash box was kept on the table at which participants were seated, to increase the motivational properties of the monetary incentives. During the break between both conditions, participants were informed about their total score in the first condition. In addition, it was repeated that they were free to choose any strategy. After task completion, most participants reported that they had made an effort to find a systematic pattern in the feedback sequences.

Participants performed equal numbers of trials in the control condition (*M* = 495 trials, *SD* = 37) and the stress condition (*M* = 490 trials, *SD* = 38; paired *t*(46) = 1.07, n.s.). The amount of money participants earned was comparable in the control (total score *M* = 45 euro cents, *SD* = 430) and the stress condition (total score *M* = 16 euro cents, *SD* = 402; paired *t*(46) = .35, n.s.). Participants reached an average end score of 61 euro cents (*SD* = 614), which was added to the €5 starting money and paid to them, at the end of the experimental session. Participants with an end score of minus €5 or less received no bonus money. Trial numbers, total scores and end scores were similar for both sexes.

### Stress induction

In order to induce a stressful state, participants were exposed to a noise stressor. This stressor consisted of discontinuous white noise of varying intensity (75–95 dB(A), 0–10 kHz), produced at our department. It included both noise intervals and inter-noise (silence) intervals. The length of each noise interval varied from 2 to 7 seconds, during which the intensity of noise varied between 75 and 95 dB(A). The length of inter-noise intervals also varied from 2 to 7 seconds. Half of the noise intervals were followed by an inter-noise interval, whereas the other half were followed by another noise interval. An inter-noise interval was never followed by another inter-noise interval. The length and intensity of noise intervals and the length of inter-noise intervals were randomly determined. The noise was played from a compact disc, and delivered by two loudspeakers in stereo mode placed on either side of the computer screen. Acute noise exposure is a common stressor, which activates the HPA axis and the sympathetic nervous system, leading to increases of stress hormones including epinephrine, norepinephrine and cortisol [40]. Moreover, acute noise exposure has been shown to impair cognitive functioning on novel and complex tasks [41], [42].

The subjective effects of exposure to the noise stressor were investigated in a pilot experiment. Participants were randomly assigned to either a silence condition (*n* = 19) or a noise condition (*n* = 17). Immediately before and after task performance, participants filled in the shortened Dutch version of the Profile of Mood States [43]. Participants in the noise group showed a significantly larger decrease in vigor (*M* = −3.4, *SD* = 3.4) relative to those in the silence group (*M* = −0.8, *SD* = 3.7; *t*(34) = −2.17, *p* = .019, one-tailed). In addition, they reported an increase in tension (*M* = +0.6, *SD* = 1.5), while the silence group reported a decrease in tension (*M* = −0.4, *SD* = 2.0; *t*(34) = 1.69, *p* = .050, one-tailed). These results confirm that exposure to the discontinuous white noise of varying intensity elicits stress in participants.

### Electrophysiological recording and data reduction

EEG was measured using 28 Sn electrodes attached to an electrocap (ElectroCap International Inc., Eaton, Ohio, USA), positioned according to the 10–10 system. Recordings were taken from channels FP1, FP2, AFz, F7, F3, Fz, F4, F8, FT7, FC3, FCz, FC4, FT8, T7, C3, Cz, C4, T8, P7, P3, Pz, P4, P8, PO7, O1, Oz, O2 and PO8, and referenced to the computed average of both mastoids. Horizontal electro-oculogram (EOG) was recorded bipolarly using two electrodes placed at the outer canthi of both eyes. Vertical EOG was measured using two electrodes placed above and below the left eye. All electrode impedances were kept below 5 kΩ. EEG and EOG signals were recorded with a 2000-Hz sample rate, a 0.16-Hz high-pass filter and a 200-Hz low-pass filter.

Off-line, EEG and EOG data were down-sampled to 256 Hz, after additional filtering with a low-pass filter of 30 Hz and a slope 48 dB/oct, for the ERP analysis only. For the ERP analysis, data were segmented in 1000-ms epochs, starting 100 ms before feedback onset. For the time-frequency analysis, segments covered 3000 ms, starting 1000 ms before feedback onset. Epochs with too rapidly changing activity (maximal allowed voltage step ±60 µV) were rejected. After removal of these artifacts, EEG was corrected for eye movements and blinks using the regression procedure of Gratton et al. [44]. Then, epochs which contained EEG voltage differences exceeding 200 µV, or EEG amplitudes exceeding +/− 100 µV, were eliminated. After these ocular correction and artifact rejection procedures, EEG was averaged relative to a 100 ms pre-feedback baseline. For the ERP analysis, separate averages were calculated for each combination of valence (gain vs. loss), magnitude (large vs. small), and stress induction (stress vs. control), resulting in eight average waveforms for each electrode and participant. For exploratory intersite phase synchronization analyses, preprocessed EEG data were converted to current source density (CSD) using the methods of Kayser and Tenke [45]. CSD estimates are based on the second spatial derivative of voltage between nearby electrode sites, acting as a reference-free, spatially enhanced signal representation. This CSD transformation accentuates local electrical activities at the expense of diminishing the representation of distal activities [46]. Thus, applying a CSD filter increases spatial selectivity and minimizes volume conduction effects.

Time-frequency analyses were performed with the Matlab-based FieldTrip toolbox [47]. To study the oscillatory dynamics of the EEG, single-trial feedback-locked data were convolved with a family of complex Morlet wavelets. These wavelets contained a fixed number of cycles of sinusoidal oscillations for each frequency band (4–7 Hz, 5 cycles; 8–12 Hz, 6 cycles; 13–20 Hz, 7 cycles; 21–30 Hz, 7 cycles). This analysis produced raw power estimates for each time point between 400 ms pre-feedback and 1000 ms post-feedback (in 10-ms steps) at frequencies of 4–30 Hz (in 0.5-Hz steps). Subsequently, a single-trial relative baseline correction was applied, in which each power value was divided by the average power of the pertaining frequency in the −400–−200 ms pre-feedback interval [48]. Then, we calculated the average power in each of the three frequency bands, for each combination of valence, magnitude and stress induction, for each participant. This single-trial approach to baseline correction has two advantages. First, it is less sensitive to the presence of noisy trials relative to classical baseline correction methods [48]. Second, it allows one to focus on phasic effects. Any tonic differences in signal between the stress induction conditions or between the sexes would also influence the baselines. By dividing by the single-trial baseline power values we corrected for tonic differences and were able to focus on phasic differences in the feedback-related interval. To evaluate tonic differences in power, we checked whether baseline power values differed between stress induction conditions and sexes. Therefore, we calculated the average absolute power in the baseline interval (−400–−200 ms pre-feedback), for each of the three frequency bands, for each stress induction condition, for each participant.

Intersite phase synchrony (ISPS) represents the extent to which phase angle differences between electrodes are consistent over trials at each time-frequency point [49]. To confirm the importance of theta-band activity in communicating the need for increased cognitive control between the MFC and the lateral PFC, we explored ISPS between FCz and F3/F4. Therefore, we ran time frequency analyses producing estimates of phase angles for each time point between 400 ms pre-feedback and 1000 ms post-feedback (in 10-ms steps) at frequencies of 4–7 Hz (in 0.5-Hz steps). Subsequently, we ran connectivity analyses for channel combinations FCz and F3, and FCz and F4. Then, a condition-specific baseline correction was applied: from each ISPS value in the feedback-related interval the average ISPS value of the pertaining frequency in the −400–−200 ms pre-feedback interval was subtracted, for each participant and condition.

### Data analysis

#### Behavioral measures

To investigate the influence of previous outcomes on current behavior, mean reaction times (RTs) and stay/switch percentages were computed as a function of the outcome on the previous trial (+/− 5 or 25 euro cents). On stay trials, participants selected the card on the same side as on the previous trial, whereas on switch trials, they chose the card on the other side. Behavioral data were analyzed using repeated measures analysis of variance (ANOVA) with the within-subjects factors valence (gain vs. loss), magnitude (large vs. small), and stress induction (stress vs. control), and the between-subjects factor sex (male vs. female). Whenever necessary, additional analyses were conducted to elucidate significant interactions. For post-hoc tests, adjustment for multiple comparisons was applied using the Bonferroni method.

#### ERPs

**Figure 1:**
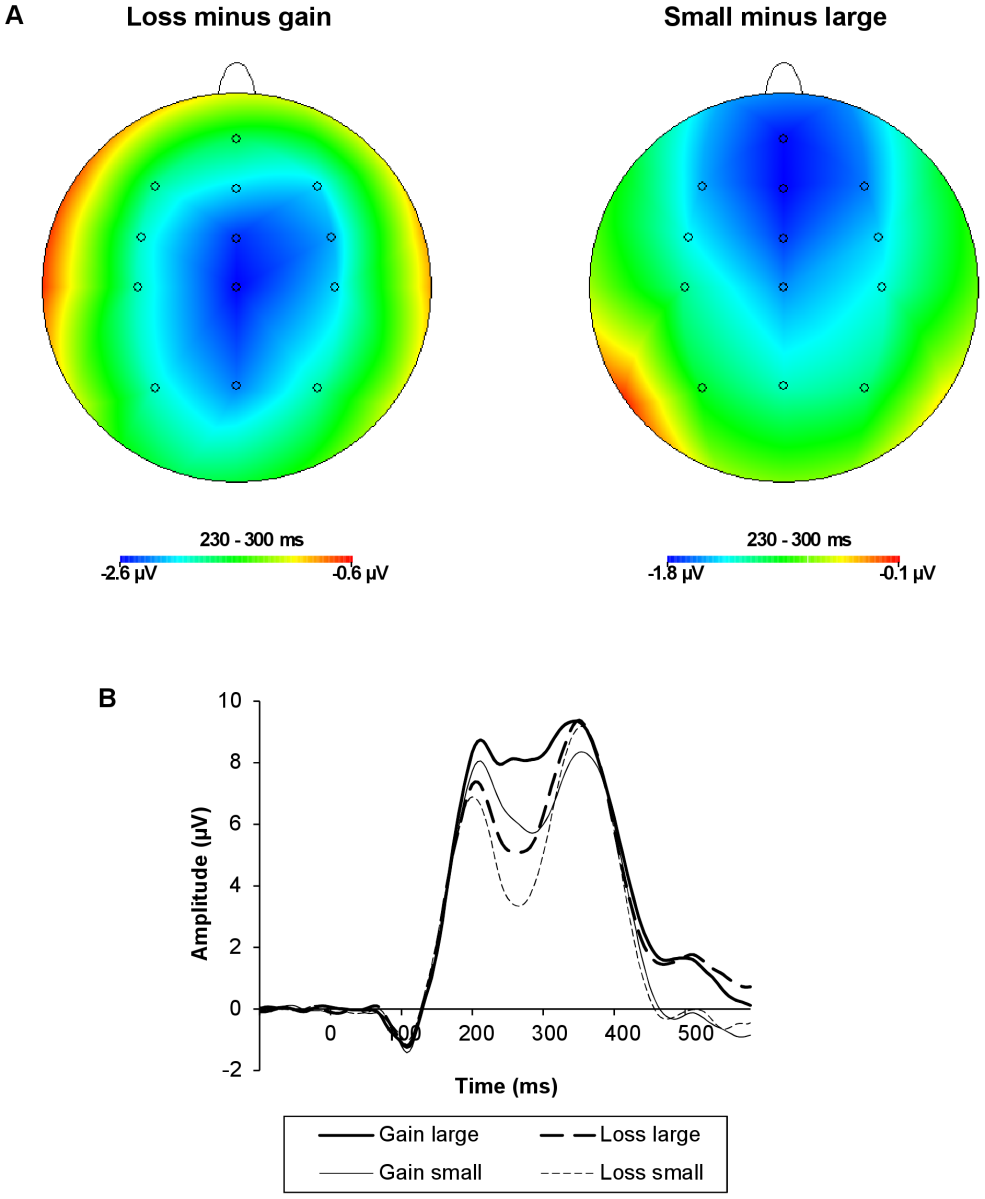
Topographical voltage maps and ERPs from FCz as a function of feedback valence and magnitude. (A) Topographical voltage maps (230–300 ms post-feedback) of the difference between loss and gain trials (left) and the difference between small and large outcome trials (right). (B) ERPs: The solid lines represent gain trials; the dashed lines represent loss trials. Thick lines represent large outcome trials; thin lines represent small outcome trials. The FRN was more negative in response to losses compared to gains, and in response to small relative to large outcomes.

For the feedback-related ERP analyses and oscillatory analyses, we focused on data from channel FCz, which is consistent with previous studies using frontocentral electrodes for these analyses (see [30]; Fig. 1). In our previous study, the FRN was measured in three different ways [26]. In order to be able to compare current FRN results with the previous results, we used the same FRN measures. First, the FRN was quantified as the mean amplitude in the 230–300 ms post-feedback interval, which is in line with previous studies (e.g., [17], [31], [50]). Second, the FRN was measured base-to-peak, which is also common practice (e.g., [51], [52]). For this purpose, we identified the most positive value within the 150–230 ms post-feedback window and, subsequently, the most negative value within a window extending from this maximum to 330 ms post-feedback. The base-to-peak FRN was quantified as the difference between these most positive and most negative values. Third, the FRN was measured as the difference in voltage between the 230–300 ms mean amplitude and the average of the mean amplitudes of the preceding (180–225 ms window) and following (320–390 ms window) peaks. Subsequently, these three FRN measures were each subjected to repeated measures ANOVAs with the within-subjects factors valence, magnitude and stress induction, and the between-subjects factor sex. Post-hoc, we ran repeated measures ANOVAs for both sexes separately, in order to elucidate divergent findings with regard to stress induction effects between the current study and our previous study [26].

**Figure 2:**
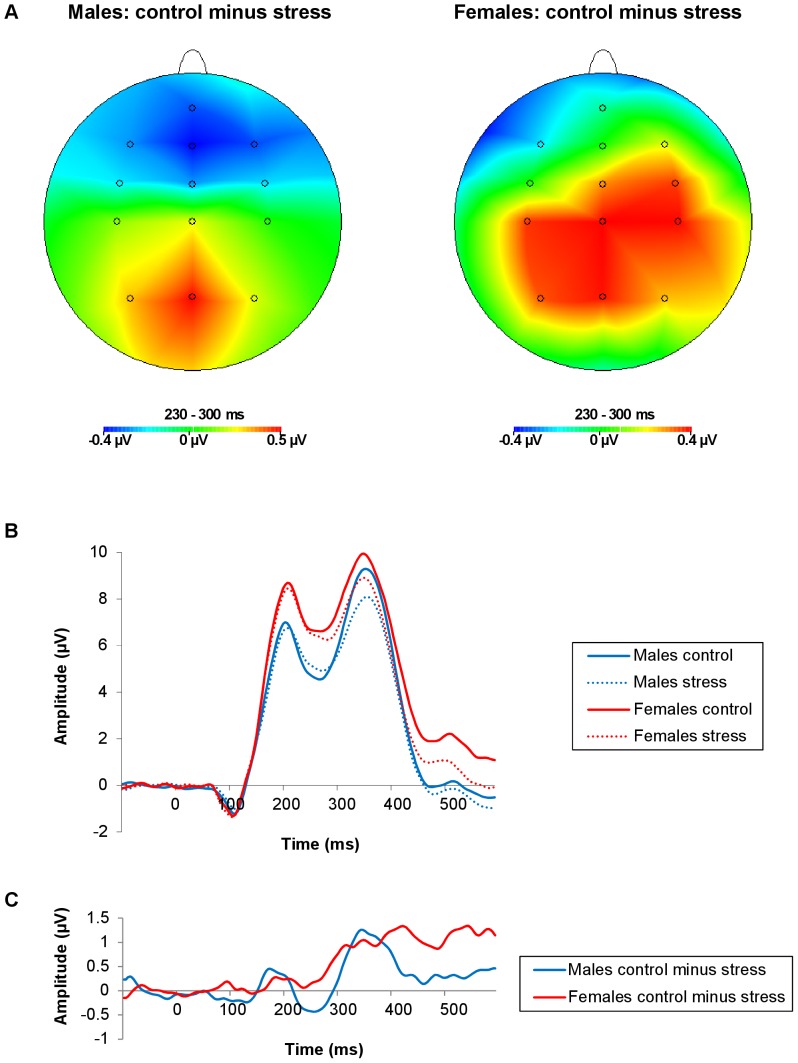
Topographical voltage maps and ERPs from FCz as a function of stress induction and sex. (A) Topographical voltage maps (230–300 ms post-feedback) of the difference between control condition and stress condition trials, for males (left) and females (right), separately. (B) ERPs: The solid lines represent control condition trials; the dotted lines represent stress condition trials. The blue lines represent males; the red lines represent females. (C) ERP difference waves of control minus stress condition trials, for males (blue line) and females (red line). The FRN amplitude was smaller in the stress relative to the control condition, but only as quantified by the mean amplitude (230–300 ms post-feedback) corrected for both preceding (180–225 ms) and following (320–390 ms) components. Sex did not modulate the FRN significantly.

Furthermore, visual inspection of the ERPs (Fig. 2) indicated that the P300 was affected by stress induction as well. As P300 amplitude might influence findings with regard to the FRN as quantified by the mean amplitude relative to preceding and following peaks, we ran post-hoc repeated measures ANOVAs on the P300. The posterior P300 was quantified as the mean amplitude at Pz, in the 300–400 ms post-feedback interval, which is in accordance with previous studies [53]. In addition, as effects on the peak following the FRN (320–390 ms post-feedback, at FCz) diverged from effects on the posterior P300, we also analyzed this fronto-central P300.

#### Oscillatory power

**Figure 3:**
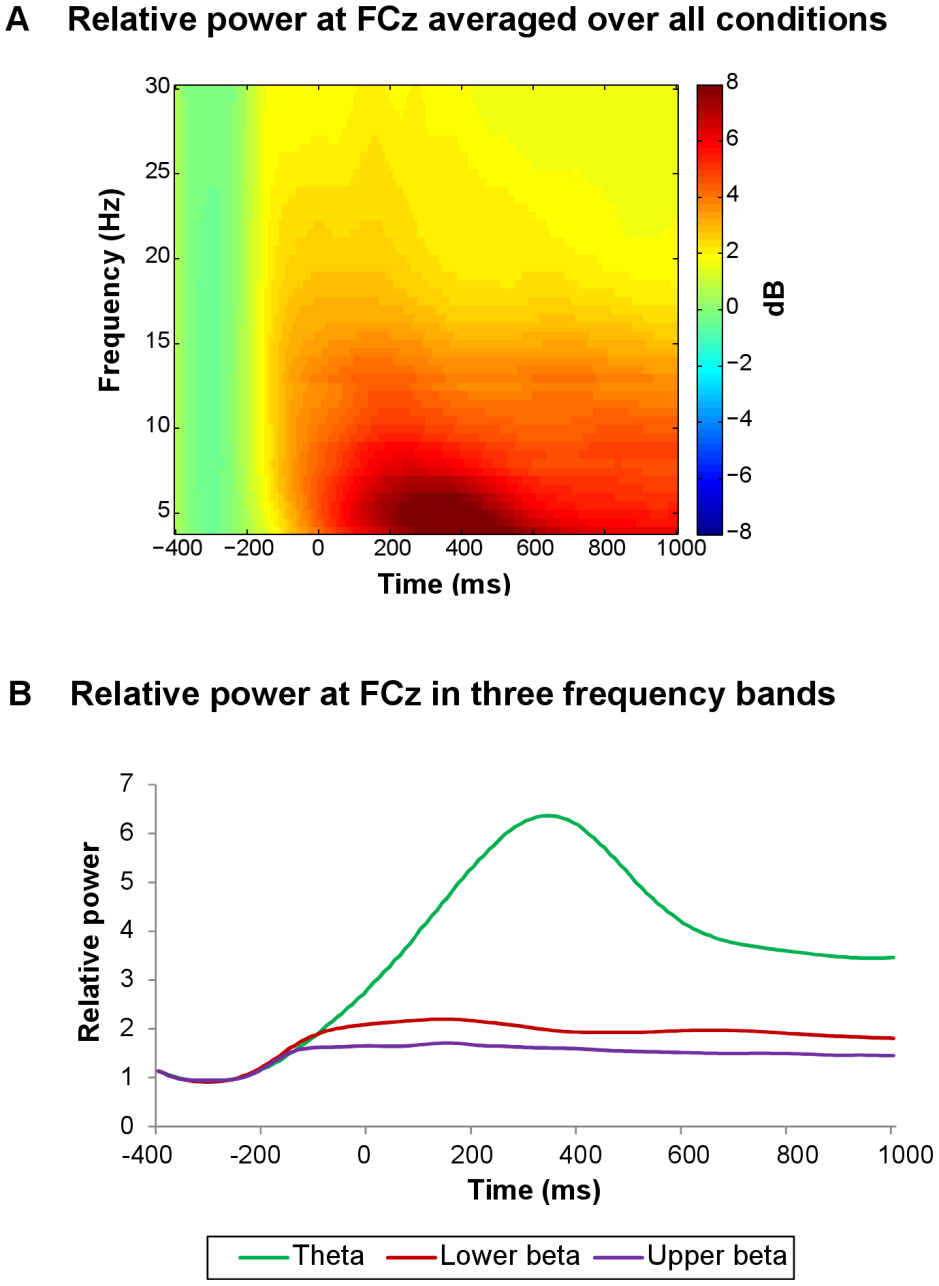
Time-frequency plot and line plots of relative power in different frequency bands, averaged over all conditions. (A) Time-frequency representation of relative power at FCz averaged over all conditions. Only for time-frequency plots, relative power averages were converted to a decibel (dB) scale, enabling comparison between different frequencies. (B) Line plots of relative power at FCz in the theta-band (4–7 Hz), lower beta-band (13–20 Hz), and upper beta-band (21–30 Hz), averaged over all conditions.

Time windows of frequency bands were selected on the basis of average power plots across all eight conditions and across all participants, at FCz (Fig. 3). Theta (4–7 Hz) was quantified as the mean activity in a 200–500 ms post-feedback window; while both lower beta (13–20 Hz) and upper beta (21–30 Hz) were measured in an early (0–300 ms) as well as a late (300–600 ms) post-feedback window, which is in line with previous studies (e.g., [25]). The resulting power values were analyzed using repeated measures ANOVA with the within-subjects factors valence, magnitude and stress induction, and the between-subjects factor sex. In addition, we examined whether power values differed in the baseline, between stress induction conditions and sexes. Average absolute baseline power values were subjected to repeated measures ANOVAs with the within-subjects factor stress induction, and the between-subjects factor sex. Finally, we performed post-hoc analyses to investigate whether significant valence and magnitude effects on feedback-related changes in oscillatory power were associated with significant valence and magnitude effects on behavioral measures, respectively. Therefore, we calculated Pearson correlation coefficients between the pertaining effects.

*Exploratory analyses: Theta-band intersite phase synchrony*: Theta-band ISPS was quantified as the mean ISPS value in a 200–500 ms post-feedback window. Theta-band ISPS was explored between medial frontal (FCz) and lateral prefrontal (F3, F4) sites. The ISPS values were analyzed using repeated measures ANOVA with the within-subjects factors valence, magnitude and stress induction, and the between-subjects factor sex.

## Results

### Behavioral results

**Figure 4:**
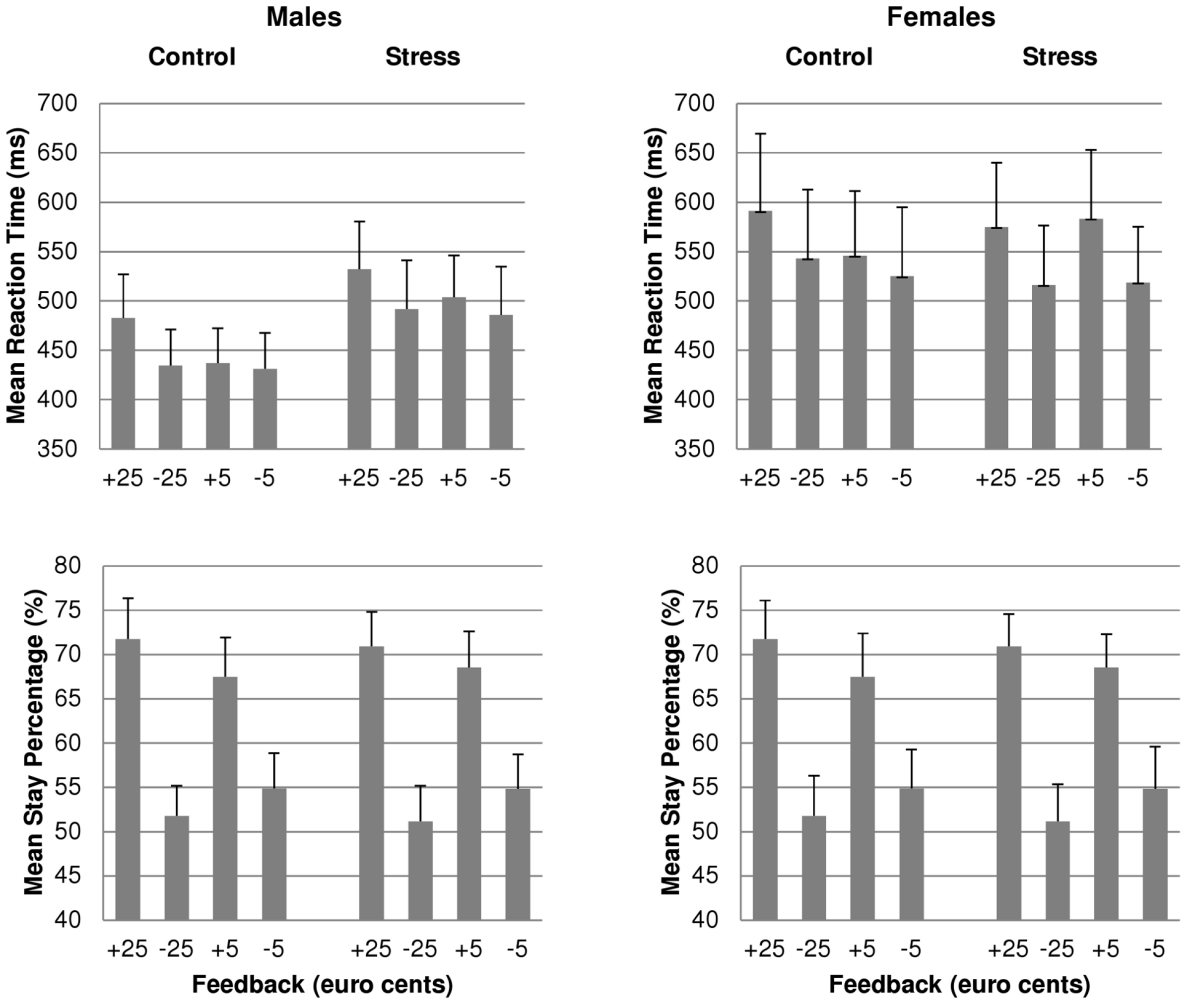
Behavior as a function of feedback type and stress induction, for males and females, separately. Mean reaction times and mean stay percentages as a function of feedback valence and magnitude, and stress induction, for males (left) and females (right), separately. Error bars represent standard errors. Participants showed longer RTs after gain than after loss trials, and after large magnitude compared to small magnitude trials. In addition, participants were more likely to repeat their card choice of the previous trial, after gains than after losses, especially after large outcomes. Neither stress induction nor sex affected behavior significantly.

Participants could win or lose either 5 or 25 euro cents, on each trial. Unbeknownst to the participants, there was no strategy they could learn to maximize their gains and minimize their losses. Despite feedback being presented in random order and thus not related to choices made, participants' behavior indicated that they were sensitive to the outcomes of their choices (Fig. 4). Participants showed longer RTs after gain trials than after loss trials (*F*(1, 45) = 20.73, *p*<.001), and after large magnitude compared to small magnitude trials (*F*(1, 45) = 4.58, *p* = .038). In addition, participants were more likely to repeat their card choice of the previous trial, after gains than after losses (*F*(1, 45) = 42.67, *p*<.001; Fig. 4), especially after large outcomes (valence by magnitude: *F*(1, 45) = 4.84, *p*<.033; large: *F*(1, 45) = 35.69, *p*<.001; small: *F*(1, 45) = 35.09, *p*<.001). Neither stress induction nor sex affected RTs or stay percentages significantly.

### ERP results

#### FRN

Table 1 summarizes the results of the repeated measures ANOVAs on the three FRN measures. The FRN was more negative in response to losses compared to gains, and in response to small relative to large outcomes (Fig. 1). These valence and magnitude effects were significant for all three FRN measures. Stress induction had a significant effect on the FRN, but only as quantified by the mean amplitude corrected for both surrounding peaks measure (Fig. 2). The FRN was smaller in the stress relative to the control condition. Sex did not modulate the FRN significantly.

**Table 1:** Summary of effects on three different FRN measures.

FRN measure	Mean amplitude (MA)		MA corrected for both peaks^1^		Base-to-peak	
Effect	*F*	*p*	*F*	*p*	*F*	*p*
Valence	75.70	<.001	65.71	<.001	30.59	<.001
Magnitude	66.30	<.001	50.07	<.001	44.43	<.001
Stress induction	<1	n.s.	6.57	.014	<1	n.s.
Sex	3.27	n.s.	<1	n.s.	<1	n.s.
Stress induction by sex	1.23	n.s.	1.46	n.s.	1.68	n.s.

The *F*(1, 45)- and *p*-values are reported.

1 Mean amplitude 230–300 ms post-feedback minus average of mean amplitudes preceding and following peaks.

**Figure 5:**
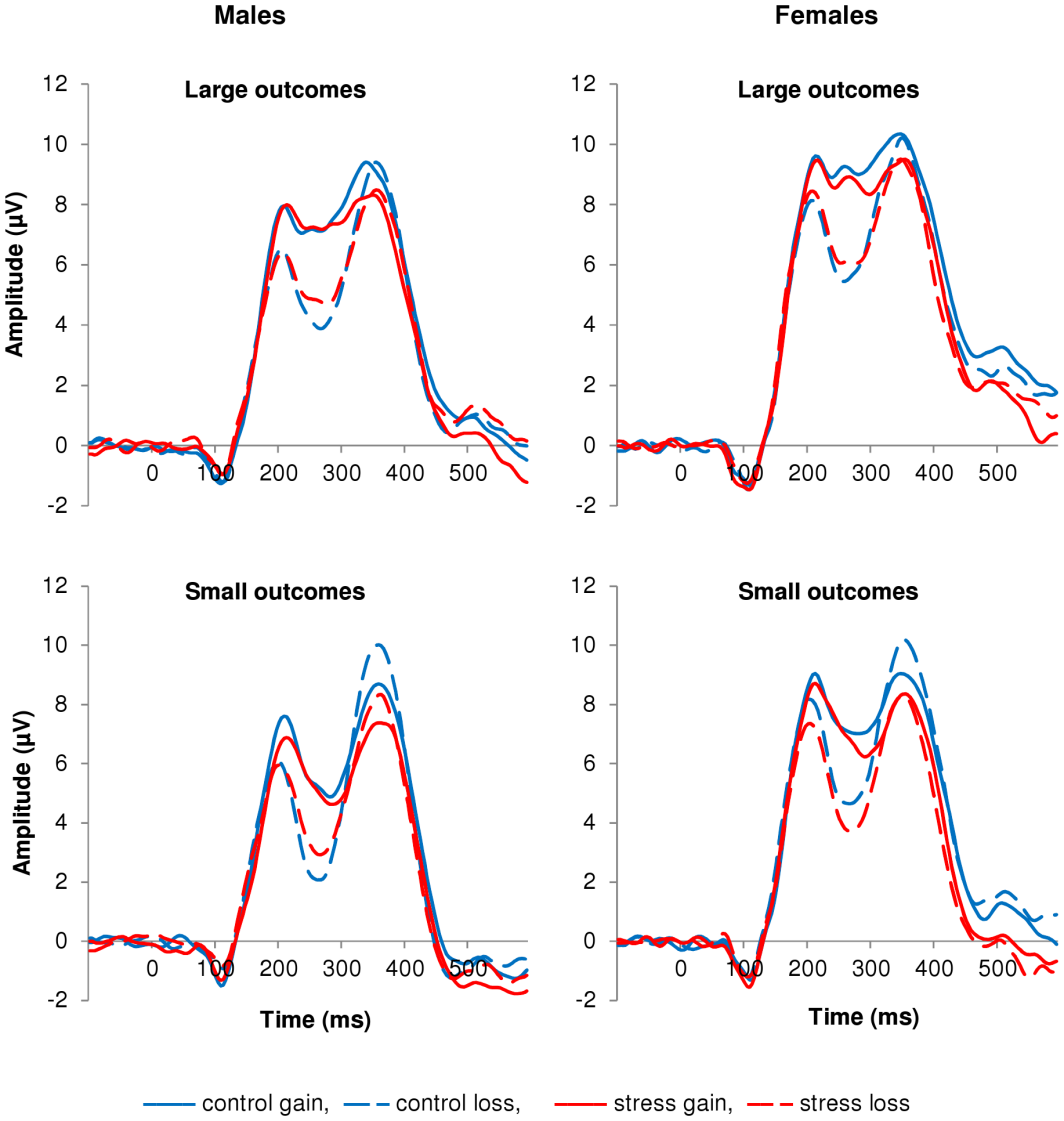
ERPs from FCz per magnitude, as a function of feedback valence and stress induction, for males and females, separately. ERPs from FCz per magnitude, as a function of feedback valence and stress induction, for males (left) and females (right), separately. The solid lines represent gain trials; the broken lines represent loss trials. The blue lines represent the control condition; the red lines represent the stress condition. The FRN amplitude was smaller in the stress relative to the control condition, but only as quantified by the mean amplitude (230–300 ms post-feedback) corrected for both preceding (180–225 ms) and following (320–390 ms) peaks. Interactions involving valence, magnitude, stress induction and sex did not reach significance.

Figure 5 shows the grand average ERPs per magnitude, as a function of valence and stress induction, for males (left) and females (right). Visual inspection suggests that valence had a smaller effect on the FRN in the stress relative to the control condition, for both large and small outcomes, in males, and for large but not small outcomes, in females. However, interactions involving valence, magnitude, stress induction and sex did not reach significance (for all three FRN measures and for all comparisons: *F*(1, 45)≤2.63, n.s).

In our previous study [26], where only male participants were included, we found a significant valence by stress induction interaction on the mean amplitude measure, and a significant magnitude by stress induction interaction on the base-to-peak measure, which we did not find in the current study. In order to clarify these divergent findings with regard to stress induction effects, we performed repeated measures ANOVAs on the pertaining measures, for both sexes separately. Neither of the two mentioned interactions were significant, although the analyses did reveal a few trends. The repeated measures ANOVAs on the mean amplitude measure showed a nonsignificant valence by stress induction interaction in males (*F*(1, 23) = 3.09, *p* = .092) and a nonsignificant valence by magnitude by stress induction interaction in females (*F*(1, 22) = 3.80, *p* = .064). The repeated measures ANOVAs on the base-to-peak measure showed nonsignificant magnitude by stress induction interactions in both sexes (both males and females: *F*<1, n.s.).

#### P300

Table 2 summarizes the results of the repeated measures ANOVAs on the posterior P300 and the fronto-central P300, respectively. The posterior P300 was more positive in response to gains relative to losses, and in response to large compared to small outcomes. The magnitude effect on the posterior P300 was present for both gains (*F*(1, 45) = 39.05, *p*<.001) and losses (*F*(1, 45) = 8.52, *p* = .005), but more pronounced for gain trials. In addition, the posterior P300 amplitude was smaller in the stress relative to control condition, but this effect was only significant for small outcomes (*F*(1, 45) = 7.19, *p* = .010), not for large outcomes (*F*(1, 45) = 2.34, *p* = .134).

**Table 2:** Summary of effects on the posterior P300 (Pz) and the fronto-central P300 (FCz).

P300 measure	Posterior P300		Fronto-central P300	
Effect	*F*	*p*	*F*	*p*
Valence	25.22	<.001	< 1	n.s.
Magnitude	33.60	<.001	13.30	.001
Valence by magnitude	7.97	.007	9.73	.003
Stress induction	4.53	.039	3.42	.071
Magnitude by stress induction	12.10	.001	5.77	.020

The *F*(1, 45)- and *p*-values are reported.

The fronto-central P300 was more positive in response to large relative to small outcomes, but only for gains (*F*(1, 45) = 21.70, *p*<.001) not for losses (*F*(1, 45) = 1.35, *p* = .251). In addition, the fronto-central P300 was smaller in the stress relative to the control condition, but this effect was only significant for small outcomes (*F*(1, 45) = 5.41, *p* = .025), not for large outcomes (*F*(1, 45) = 1.66, n.s.).

### Oscillatory power results

**Figure 6:**
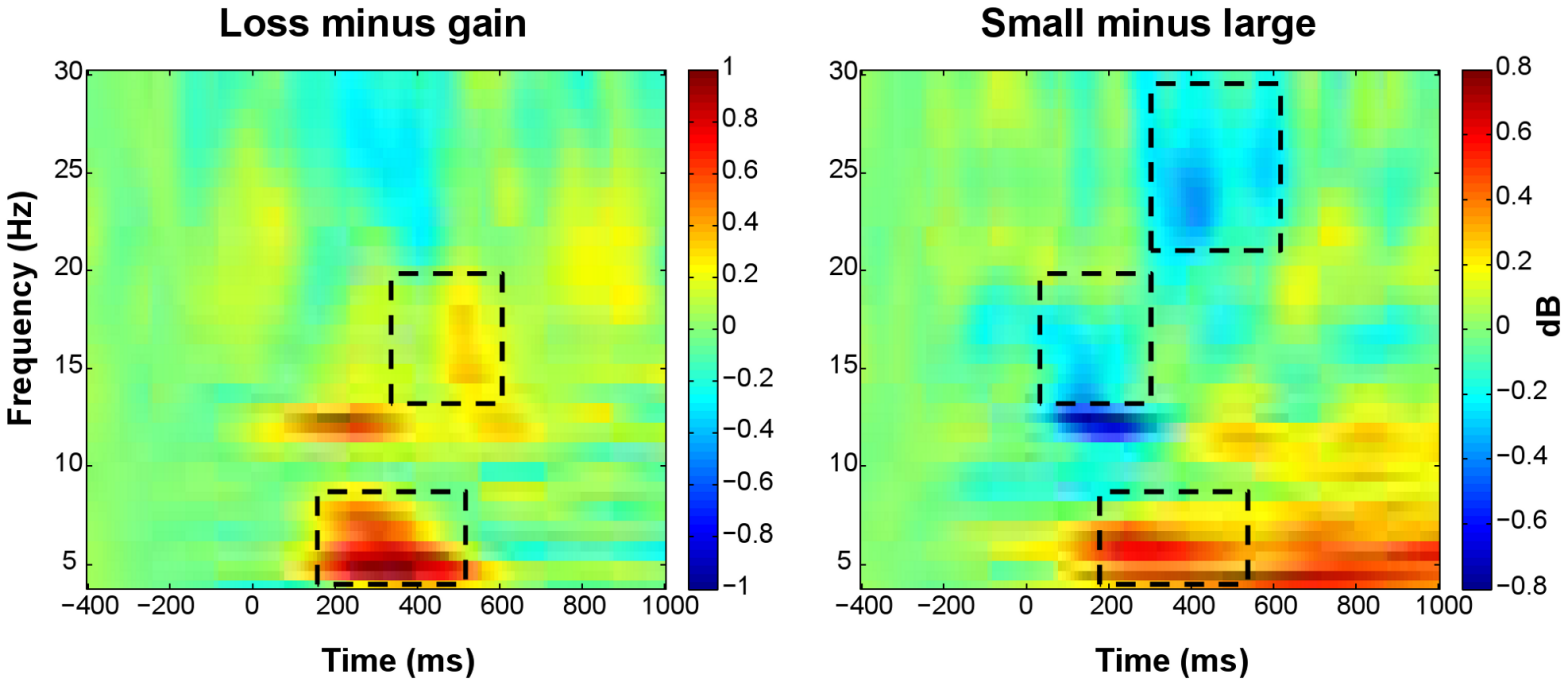
Time-frequency plots showing effects of feedback valence and magnitude. Time-frequency representations of the difference between loss and gain trials (left), and of the difference between small and large outcome trials (right). The plots show relative power (dB) at FCz. Only for time-frequency plots, relative power averages were converted to a decibel (dB) scale, enabling comparison between different frequencies. Line boxes highlight larger increases in theta and late lower beta-band power for losses relative to gains (left); larger increases in theta power and smaller increases in early lower beta-band and late upper beta-band power for small compared to large outcomes (right).

**Figure 7:**
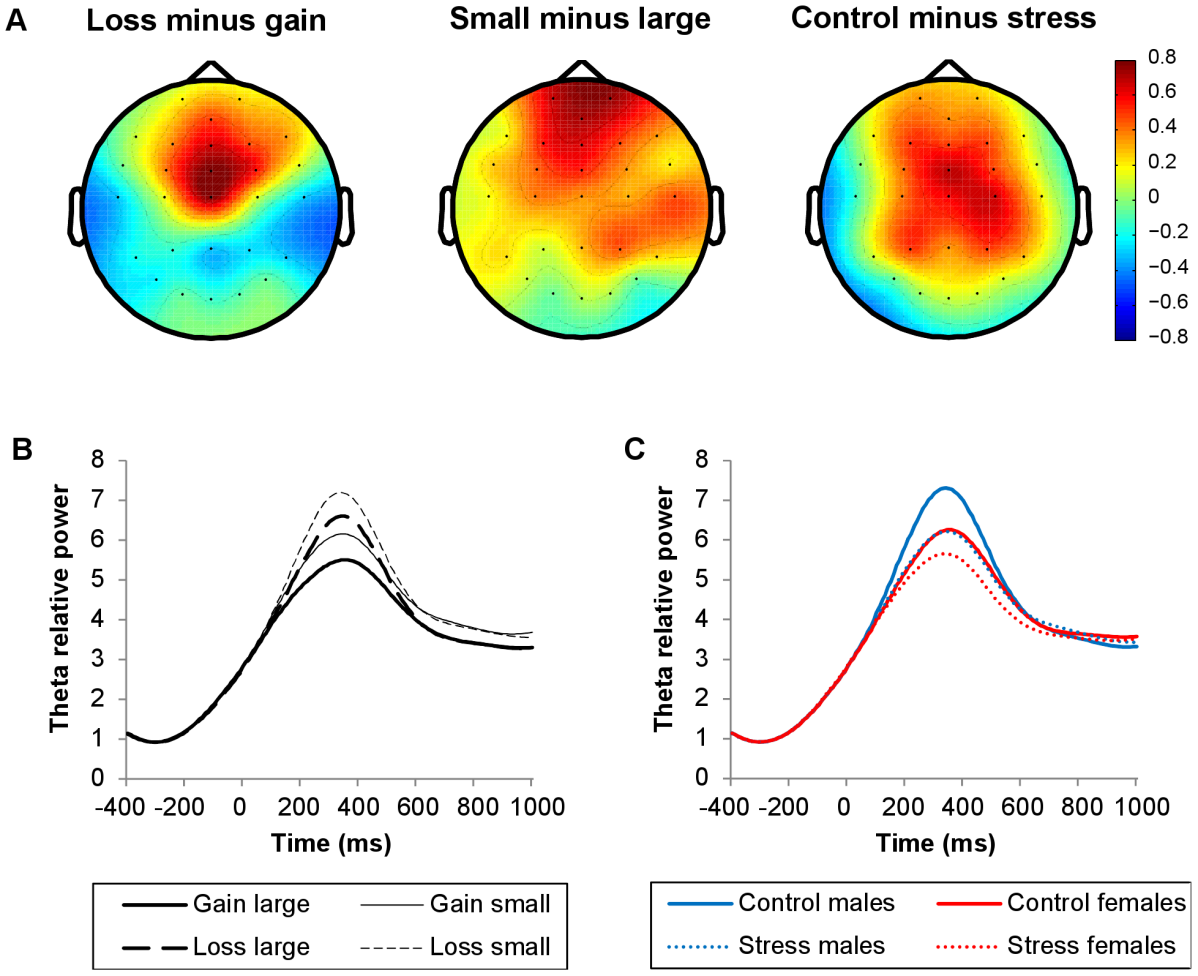
Topographical maps and line plots of theta relative power. Plots of theta relative power (4–7 Hz, 200–500 ms post-feedback). (A) Topographical maps of the difference between loss and gain trials, the difference between small and large outcome trials, and the difference between control condition and stress condition trials. (B) Line plots of theta relative power at FCz as a function of valence and magnitude. (C) Line plots of theta relative power at FCz as a function of stress induction and sex. Theta power increases were larger following losses versus gains, small versus large outcomes, and in the control versus stress condition. Sex did not modulate theta power significantly.

**Figure 8:**
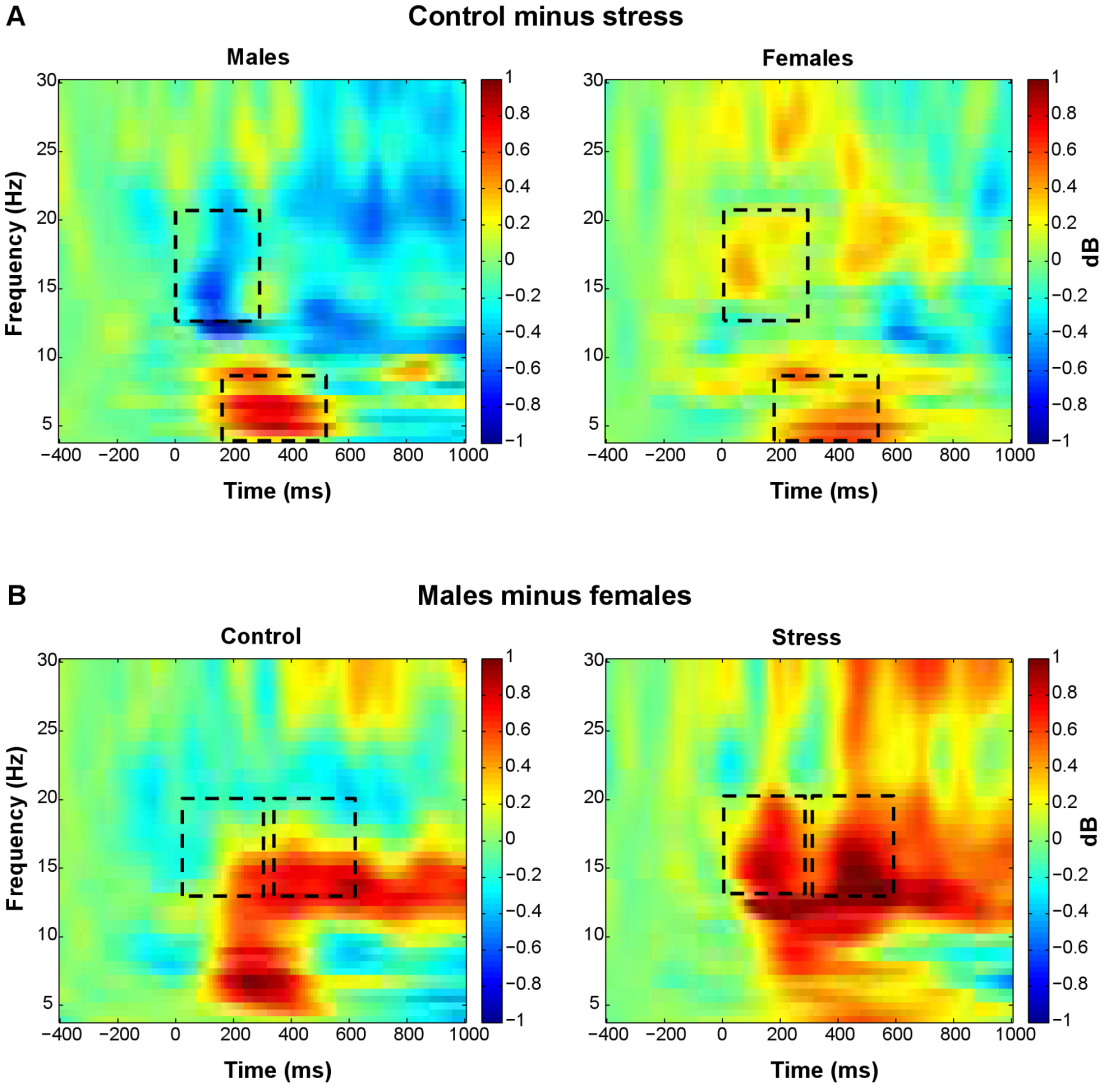
Time-frequency plots showing stress induction by sex interaction. (A) Time-frequency plots for the difference between control and stress trials, for males (left) and females (right). (B) Time-frequency plots for the difference between males and females, in control trials (left) and stress trials (right). The plots show relative power (dB) at FCz. Only for the time-frequency plots, relative power averages were converted to a decibel (dB) scale, enabling comparison between different frequencies. Line boxes highlight larger theta power increases in the control relative to the stress condition in both sexes. Males only showed an effect of stress induction on early lower beta-band power, approaching significance (*p* = .053), with larger increases in the stress relative to the control condition. More pronounced increases in lower beta power were observed in males than in females. In the early interval, this sex difference was restricted to the stress condition, whereas in the late interval, this difference was observed for both conditions.

Theta power and both early (0–300 ms post-feedback) as well as late (300–600 ms) lower and upper beta-band power increased after all feedback types, in both stress induction conditions, relative to a pre-feedback baseline interval (Fig. 3). The observed theta power increase was larger for losses than gains, and for small relative to large outcomes (valence: *F*(1, 45) = 15.37, *p*<.001; magnitude: *F*(1, 45) = 19.70, *p*<.001; Fig. 6, Fig. 7). In addition, the increase was more pronounced in the control compared to the stress condition (*F*(1, 45) = 7.26, *p* = .010; Fig. 7, Fig. 8). Sex did not modulate theta power.

**Figure 9:**
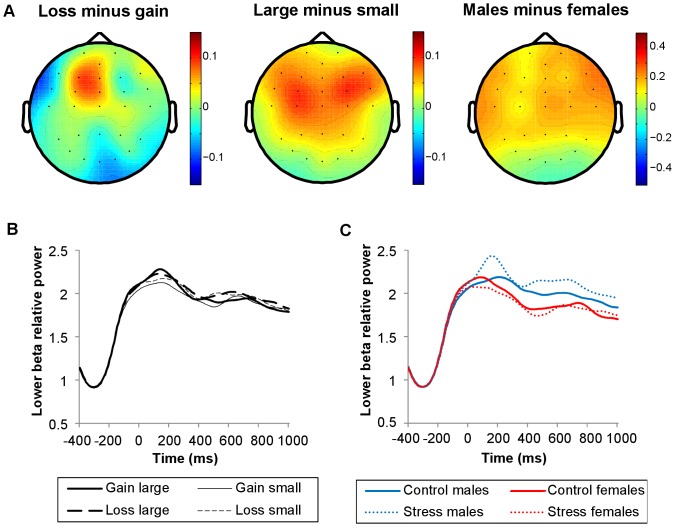
Topographical maps and line plots of lower beta-band relative power. Plots of lower beta-band relative power (13–20 Hz). (A) Topographical maps of the difference between loss and gain trials (300–600 ms post-feedback), the difference between large and small outcome trials (0–300 ms), and the difference between males and females (0–600 ms). (B) Line plots of lower beta-band relative power at FCz as a function of valence and magnitude. (C) Line plots of lower beta-band relative power at FCz as a function of stress induction and sex. Lower beta-band power increases were larger following losses than gains (300–600 ms), and larger for large relative to small outcomes (0–300 ms). More pronounced increases in lower beta power were observed in males than in females. In the early interval, this sex difference was restricted to the stress condition, whereas in the late interval, this difference was observed for both conditions.

Early lower beta power was more pronounced for large relative to small outcomes (*F*(1, 45) = 4.57, *p* = .038; Fig. 6, Fig. 9). In addition, early lower beta power depended on the combination of stress induction condition and sex (stress induction by sex: *F*(1, 45) = 6.22, *p* = .016: Fig. 8, Fig. 9). Both sexes showed similar power increases in the control condition, while in the stress condition, males showed larger power increases than females (sex effect in stress condition: *F*(1, 45) = 6.68, *p* = .013). Separate analyses for both sexes revealed an effect of stress induction, in males only, with larger power increases in the stress relative to the control condition, approaching significance (stress induction effect in males: *F*(1, 23) = 4.18, *p* = .053). Late lower beta power was larger for losses relative to gains (*F*(1, 45) = 4.29, *p* = .044; Fig. 6, Fig. 9). In this late interval, males showed larger increases in lower beta power compared to females, in both stress induction conditions (sex: *F*(1, 45) = 6.99, *p* = .011; stress induction by sex: *F*(1, 45) = 3.24, n.s; Fig. 8, Fig. 9).

**Figure 10:**
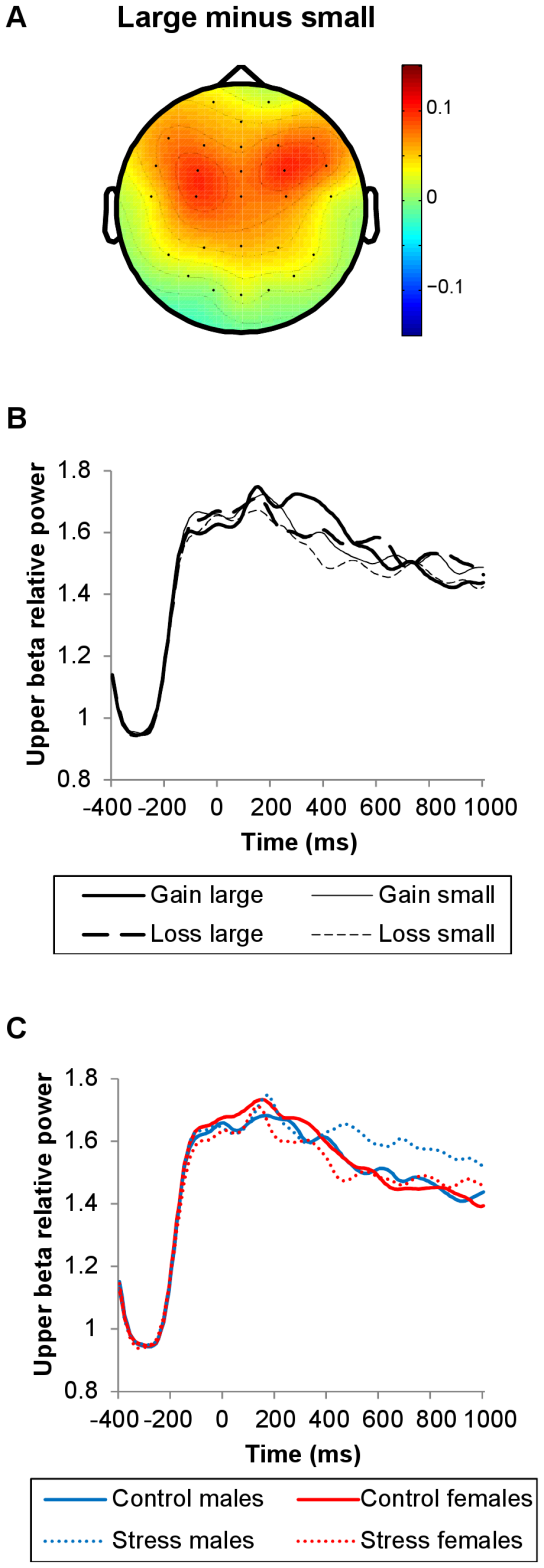
Topographical maps and line plots of upper beta-band relative power. Plots of upper beta-band relative power (21–30 Hz). (A) Topographical map of the difference between large and small outcome trials (300–600 ms post-feedback). (B) Line plots of upper beta-band relative power at FCz as a function of valence and magnitude. (C) Line plots of upper beta-band relative power at FCz as a function of stress induction and sex. Upper beta-band power increases were larger for large relative to small outcomes (300–600 ms). Neither stress induction nor sex modulated upper beta-band power significantly.

Whereas lower beta power was modulated by feedback magnitude in the early interval, upper beta power was modulated by feedback magnitude in the late interval. Similar to early lower beta power, late upper beta power was more pronounced for large relative to small outcomes (*F*(1, 45) = 5.63, *p* = .022; Fig. 6, Fig. 10). Neither stress induction nor sex influenced upper beta power (Fig. 8, Fig. 10).

Furthermore, we examined whether absolute power values differed in the baseline interval (−400–−200 ms pre-feedback), between stress induction conditions and sexes. Neither stress induction nor sex modulated theta baseline power. However, sex modulated lower beta baseline power, with larger power values for females relative to males (*F*(1, 45) = 5.21, *p* = .027), in both stress induction conditions. Note that in the feedback-related interval, men showed larger increases in lower beta power than women, relative to the baseline interval. In the early interval, this sex difference was present only in the stress condition, while in the late interval, this sex difference was present in both control and stress conditions. Furthermore, stress induction affected upper beta baseline power, with larger power values for the stress relative to the control condition (*F*(1, 45) = 6.78, *p* = .012), for both sexes.

Finally, we performed post-hoc analyses to investigate whether significant valence and magnitude effects on feedback-related changes in oscillatory power were associated with, significant valence and magnitude effects on behavioral measures, respectively. Effects of valence and magnitude on feedback-related theta and beta power were not significantly correlated with effects of valence and magnitude on mean RTs and mean stay percentages (Table 3).

**Table 3:** Correlations between effects on behavior and oscillatory power.

Effect on behavior	Valence effect on reaction times	Valence effect on stay percentages	Magnitude effect on reaction times
Effect on oscillatory power	*r*	*r*	*r*
Valence effect on theta power	.174	.171	n/a^1^
Valence effect on late lower beta-band power	−.052	.152	n/a
Magnitude effect on theta power	n/a	n/a	.080
Magnitude effect on early lower beta-band power	n/a	n/a	.016
Magnitude effect on late upper beta-band power	n/a	n/a	.100

Pearson's *r*(47)-values are reported (all nonsignificant).

Correlations between significant valence and magnitude effects on feedback-related changes in oscillatory power and significant valence and magnitude effects on behavioral measures, respectively.

1 Not applicable.

In short, theta power was larger following losses than gains, small compared to large outcomes, and in the control relative to the stress condition. Theta power did not depend on sex. Late lower beta-band power was larger following losses than gains. Both early lower beta and late upper beta power were larger for large relative to small outcomes. More pronounced increases in lower beta power were observed in males than in females. In the early interval, this sex difference was restricted to the stress condition, whereas in the late interval, this difference was observed for both conditions. Whereas neither stress induction nor sex affected theta baseline power, these factors differentially modulated lower and upper beta baseline power. Effects of valence and magnitude on feedback-related oscillatory power were not significantly correlated with effects on behavior.

*Exploratory results: Theta-band intersite phase synchrony*: Theta-band ISPS was significantly higher after loss trials compared to gain trials between FCz and F3 (*F*(1, 45) = 33.84, *p*<.001), and between FCz and F4 (*F*(1, 45) = 51.30, *p*<.001). Neither stress induction nor sex affected theta-band ISPS between these sites.

## Discussion

Aim of the present study was to investigate whether acute stress alters decision making by modulating feedback processing, and whether stress effects differ between men and women. In order to do so, we examined effects of feedback valence and magnitude on the feedback-related EEG response, in a control and a stress condition, in men and women. We used both ERP and time-frequency analyses, measuring the FRN and changes in theta and beta oscillatory power, respectively. During the stress condition, participants were exposed to a noise stressor. While the FRN and feedback-related theta power were similarly affected by stress induction in both sexes, feedback-related beta power depended on the combination of stress induction condition and sex. Behavior was not modulated by stress induction or sex.

Participants completed a simple gambling task, in which each choice was followed by feedback indicating the amount of money won or lost on that trial. They were instructed to earn as much money as possible, but as gains and losses were assigned randomly, there was no strategy they could learn to optimize their monetary results. Nevertheless, participants' choice behavior indicated that they actually were sensitive to the valence and magnitude of previous outcomes. They were, for example, more likely to repeat their previous choice, if that choice had resulted in a gain than if that choice had resulted in a loss, indicating that they took previous outcomes into account, in their decisions. This is in line with the idea that decision makers, when faced with uncertainty, actively search for information to improve future choices [54].

### Effects of feedback valence and magnitude

The effects of feedback valence and magnitude on the FRN and feedback-related theta power showed a consistent pattern. Both the FRN and theta power were larger for losses compared to gains, and for small relative to large outcomes, which is in line with previous studies investigating the effects of valence and/or magnitude on these measures (FRN, both valence and magnitude: [32], [55], [56]; theta power, valence: [27], [30], [32]; theta power, magnitude: [57]). According to the conflict monitoring theory, MFC activity – as reflected in the FRN amplitude and theta power increase – is especially high in situations of high behavioral uncertainty [23], [29]. This increased MFC activity is thought to communicate a need for increased cognitive control to the lateral PFC, which performs regulatory processes to implement adjustments [19], [24], [25]. Losses are more likely to cause a higher level of behavioral uncertainty relative to gains: decisions preceding losses were apparently wrong and require adjustments of behavior; whereas decisions preceding gains were apparently right, indicating that choice behavior was efficient. In addition, small outcomes probably generate more uncertainty than large outcomes, as their meaning is less equivalent: a small gain is a gain, but still not optimal; and while a large loss clearly points to a need for adjustments, it is less clear what to do after a small loss [26]. Our findings fit well with the uncertainty account of MFC activity, as we did observe an increase in FRN and theta power in response to losses and small outcomes relative to gains and large outcomes, respectively.

Our exploratory analyses of theta-band ISPS between the MFC and lateral PFC revealed increased ISPS after loss relative to gain trials, which is in accordance with earlier studies [25], [46]. It confirms the importance of theta oscillations in signaling a need for increased cognitive control between the MFC and the lateral PFC. Nevertheless, ISPS between these sites was not affected by magnitude, while theta power was, suggesting that connectivity and power in the theta-band can be differentially modulated by feedback properties.

The effects of valence and magnitude on feedback-related beta power differed between frequency bands and across time windows. In general, beta-band activity has been linked to the maintenance of a sensorimotor or cognitive state [33]. From this perspective, it might be expected that beta power increases are larger when the maintenance of the status quo is likely intended (e.g., after gains) than when a change is intended (e.g., after losses). Previous studies have indeed shown increased upper beta-band power over frontocentral sites in response to positive versus negative feedback or gains versus losses [25], [27], [32], [57]. In the present study, however, we could not replicate this valence effect on upper beta-band power. Moreover, for late lower beta-band activity we even found the opposite effect, that is larger power for losses than gains, indicating that this functional interpretation of beta-band activity neither holds for *lower* beta-band activity in feedback processing.

A somewhat different interpretation of the functional role of beta-band activity has been postulated by Baker [58]. With regard to motor control, he proposed that beta-band activity “may hold overt motor output constant in order to render the interpretation of the proprioceptive state more effective”. The processing of proprioceptive feedback is necessary for monitoring the status quo and recalibrating the sensorimotor system. In addition, this monitoring of the peripheral state may enable the maintenance of a constant motor output through rapid feedback corrections [58]. If beta-band activity has a similar function in cognitive processing, our findings suggest that losses relative to gains are followed by a more effective monitoring of feedback information.

In addition to feedback valence, beta-band activity was influenced by feedback magnitude. Increases in early lower beta-band power as well as late upper beta-band power were larger after large relative to small outcomes. Only a few studies, using gambling tasks, investigated the effects of feedback magnitude on beta-band activity. Marco-Pallares et al. [32] found enhanced upper beta power (20–30 Hz, 250–400 ms post-feedback) for maximum relative to minimum gains but not for losses. In a more recent study by HajiHosseini et al. [57], no effect of magnitude on beta-gamma activity (25–35 Hz, 200–400 ms) was found. Following the interpretation of Baker [58], our findings suggest that large relative to small outcomes, similar to losses versus gains, are followed by a more effective processing of feedback information. With regard to behavior, large relative to small outcomes were indeed followed by slightly slower RTs. Nevertheless, the respective magnitude effects on mean RTs and beta-band activity did not correlate.

It should be noted that effects of feedback valence and magnitude on beta-band activity were present but not maximal at FCz (see Fig. 9, Fig. 10), the electrode we chose on the basis of previous feedback processing literature [25], [27], [32], [57]. Further research is needed to clarify the functional role of beta-band activity in feedback processing, and to determine which brain areas communicate through beta oscillations during feedback processing.

### Effects of acute noise stress and sex

Stress has been shown to affect brain regions underlying feedback processing and feedback learning (see for reviews, [13], [14]). Therefore, we expected acute noise stress to modulate feedback-related brain activity in the present study. Indeed, we found that the increase in theta power in response to feedback was smaller in the stress relative to the control condition. Importantly, this stress effect on theta power was not yet present in the pre-feedback baseline interval, but specifically occurred in response to feedback. Increases in theta power are thought to signal a need for increased cognitive control in uncertain conditions [23], [25]. Therefore, the smaller increase in the stress relative to the control condition indicates that acute stress affects performance monitoring and, as a possible consequence, adjustments in cognitive control. Furthermore, stress-related theta modulations were similar for males and females, suggesting that the impact of acute stress on performance monitoring in this task does not differ between men and women in the midluteal phase of their menstrual cycle.

Based on previous studies, we expected the FRN to be affected by acute noise stress as well [26], [59]. Indeed, we found a smaller FRN in the stress relative to the control condition. However, this stress effect on the FRN was only present for the mean amplitude corrected for both peaks measure. Although the effects of valence and magnitude on the FRN were largely similar in the present study and in our previous study [26], the effects of stress induction showed dissimilarities between the two studies. In the current study, we found a significant main effect of stress induction on the mean amplitude corrected for both peaks measure, which was absent in the previous study. Visual inspection of the ERPs in our previous study did suggest an effect of stress induction on this FRN measure which seemed more pronounced for the unpredictable relative to the predictable noise stressor (see Fig. 3–6, in [26]). This stress induction by stressor type interaction suggests that the divergent findings between the current study and the previous study may be partly due to the fact that in the current study, all participants (n = 47) were exposed to the unpredictable noise stressor, whereas in the previous study, only half of the participants (n = 16) were exposed to this stressor, while the other half were exposed to the predictable stressor. However, note that this interaction did not reach significance in the previous study and was therefore not reported. In our previous article [26], we did not report the following statistics for the mean amplitude corrected for both peaks measure, as they were nonsignificant. The FRN was nonsignificantly smaller in the stress relative to the control condition (stress induction: *F*(1, 30) = 3.55, *p* = .069). This stress induction effect was nonsignificantly more pronounced for the unpredictable relative to the predictable noise stressor (stress induction by stressor type: *F*(1, 30) = 3.37, *p* = .077).

In addition, in the previous study, we found a significant valence by stress induction interaction on the mean amplitude measure, which we did not find in the current study. Visual inspection of ERPs in the present study suggested differential stress induction effects between men and women, on this measure (see Fig. 5). However, pertaining interaction effects did not reach significance. Post-hoc analyses for both sexes separately also did not yield significant interaction effects, although the valence by stress induction interaction in males approached significance. The divergent findings may be partly explained by the fact that the previous study had 32 male participants, whereas the current study had only 24 male participants, implicating reduced power in the present study.

Finally, in the previous study, we found a significant magnitude by stress induction interaction on the base-to-peak measure, which we did not find in the current study. We cannot explain this divergent finding, as the post-hoc analyses for both sexes separately showed nonsignificant interactions in both males and females. In conclusion, part of the divergent findings between the present and previous study may be explained by differences in experimental set-up (i.e., number and sex of participants, and noise stressor type). Although the findings of both studies together suggest that stress induction indeed affects the FRN, more research with larger sample sizes is evidently needed before well-founded conclusions on this matter can be drawn.

As in our previous study, we found that FRN results were dependent on the method of measuring FRN amplitude [26]. More specifically, we found that stress induction only had a significant effect on the FRN if the amplitude was computed relative to both surrounding peaks. Post-hoc analysis of the fronto-central P300 showed that the amplitude was smaller in the stress relative to the control condition, for small outcomes. Correcting for the amplitude of this fronto-central P300 yielded a main effect of stress induction on the FRN, compared to the results for the FRN measures that did not correct for this component (mean amplitude measure, base-to-peak measure). Due to possible overlap between the FRN and other ERP components, the measurement of the FRN is complex. One would like to isolate the latent neural processes underlying the FRN, but it is impossible to determine precisely which latent processes add up to any specific ERP component [60]. By correcting for the P300, one aims to eliminate neural processes that are unrelated to the FRN. Nevertheless, it remains inconclusive which correction procedure is most appropriate, as it is not clear when and where overlap between components starts and ends.

As we stated earlier, our findings with regard to the effects of feedback valence and magnitude were largely comparable across FRN and theta measures, suggesting that these measures reflect similar neural processes. Accordingly, it has been proposed that the FRN partially reflects a theta-band oscillatory process [27], [29]. Importantly, while the present stress effects were similar for the mean amplitude corrected for both peaks FRN measure and theta power, the other two FRN measures did not show stress effects. These discrepant findings between FRN measures might suggest that measuring the FRN while correcting for overlap with both surrounding components, relative to measuring the FRN while neglecting overlap with other components (mean amplitude) or correcting for the preceding component only (base-to-peak), results in a measure that better captures theta-band activity. Feedback processing and learning likely rely on large-scale brain networks which communicate through synchronized electrophysiological oscillations. As Cohen et al. [24] have discussed, conceptualizing the feedback-related EEG response as a temporal-spatial-frequency landscape of oscillatory dynamics – instead of an ERP component with one peak – enables research results to be directly related to neurophysiological phenomena, such as population-level neuronal activity.

Up till now, little is known about the effects of acute stress on oscillatory power in response to action outcomes. Nevertheless, our findings with regard to theta power – smaller feedback-related increases in the stress relative to the control condition – are in accordance with previous studies showing that acute noise stress has a deleterious effect on higher-order cognitive control functions (e.g., [41], [42]). Moreover, we found additional evidence for stress-induced modulations of feedback processing. Stress seems to impair the ability to modulate behavior as a function of past positive or negative feedback [10], [11]. In addition, stress reduces reward-related activation in the MFC [15], and in the dorsal striatum and OFC [16]. The same brain regions have been linked to the generation of feedback-related oscillations: the MFC is implicated in the generation of feedback-related theta oscillations (see [24]), while the OFC is a likely source of feedback-related beta oscillations [32].

While stress-related theta modulations were similar for both sexes, stress-related lower beta-band modulations were sex-dependent. In the stress condition, men showed larger feedback-related increases in early lower beta power than women. Men and women also showed tonic differences in lower beta-band power as revealed by the larger baseline power values for females than males, in both stress induction conditions. The stress by sex interaction only became significant after feedback presentation, indicating that stress had an additional impact on sex differences, in the feedback-related interval. These differential stress effects on feedback processing may be related to sex-specific stress effects on decision-making behavior, that have been reported in recent studies [5], [9], [34]. As feedback processing and learning are crucial to adaptive decision making, their modification will likely affect decision making. Note, however, that in the present study, these effects were not reflected in behavioral changes, possibly due to the fact that participants could not learn a strategy to improve their performance.

Abnormal feedback processing is regarded as a causal factor in the pathogenesis of particular stress-related disorders [3], [4]. Depression, for example, is characterized by negative mood and anhedonia, that is loss of the ability to experience pleasure from normally rewarding stimuli. Neurophysiological studies have reported enhanced [61] as well as blunted [62] responses to feedback in depressive patients, these opposite findings being ascribed to differences in illness severity. Considering the sex-specificity of the stress effects on feedback processing we observed, one might argue that differences between men and women may indeed explain (at least partly) the sex-specific prevalence rates of these stress-related disorders.

In the early interval, men showed larger increases in lower beta power than women, only in the stress condition. In the late interval, this sex difference was present in both control and stress conditions, indicating that the neural underpinnings of feedback processing in general are at least partly sex-dependent. Sex differences in feedback processing may be related to sex differences in decision-making behavior. Van den Bos et al. [9] conducted a review on sex differences in performance on the Iowa Gambling Task, a decision-making task in which subjects have to learn through exploration to differentiate between long-term advantageous and long-term disadvantageous card decks. Both men and women solve this task, but women need more trials before they consistently prefer the long-term advantageous decks. On the basis of their review, the authors proposed that men focus on long-term pay off of decks, while women focus on both long-term pay off and on win-loss frequencies. They suggested that women may be more sensitive than men to occasional losses. In the present study, however, we did not find evidence for the latter.

In conclusion, we have found that acute stress impairs performance monitoring in both sexes, as reflected in changes in FRN amplitude and frontocentral theta-band power. In addition, our findings with regard to early lower beta-band power suggest that men and women show sex-dependent stress effects on feedback processing, as well. The latter effects may be related to sex-specific prevalence rates in stress-related disorders.
